# Role of myocardial strain imaging in diagnosing inducible myocardial ischemia with treadmill contrast-enhanced stress echocardiography

**DOI:** 10.1186/s12872-024-03926-8

**Published:** 2024-05-16

**Authors:** Rami M. Abazid, Nilkanth Patil, Maged Elrayes, Mark Chandy, Magdi Hassanin, Andrew Mathew, Sabe De, Rodrigo Bagur, Nikolaos Tzemos

**Affiliations:** 1 Division of Cardiology, Department of Medicine, London Health Sciences Centre, University Hospital, 339 Windermere Road, PO Box 5010, London, ON N6A 5A5 Canada; 2 Northern Ontario Medical School (NOSM) University, Department of Medicine, Sault Area Hospital, Sault Ste. Marie, Canada

**Keywords:** Myocardial strain, Treadmill, Stress Echocardiography, Contrast

## Abstract

**Introduction:**

The aim of this study is to analyze the diagnostic value of global longitudinal strain (GLS) in detecting inducible myocardial ischemia in patients with chest pain undergoing treadmill contrast-enhanced stress echocardiography (SE).

**Methods:**

We retrospectively enrolled all patients who underwent invasive coronary angiography after treadmill contrast-enhanced SE. Rest and peak-stress myocardial GLS, segmental LS, and LS of 4-chamber (CH), 2-CH, and 3-CH views were reported. Luminal stenosis of more than 70% or fractional flow reserve (FFR) of < 0.8 was considered significant.

**Results:**

In total 33 patients were included in the final analysis, among whom sixteen patients (48.4%) had significant coronary artery stenosis. Averaged GLS, 3-CH, and 4-CH LS were significantly lower in patients with critical coronary artery stenosis compared to those without significant stenosis (-17.1 ± 7.1 vs. -24.2 ± 7.2, *p* = 0.041), (-18.2 ± 8.9 vs. -24.6 ± 8.2, *p* = 0.045) and (-14.8 ± 6.2 vs. -22.8 ± 7.8, *p* = 0.009), respectively. Receiver operating characteristic (ROC) analysis of ischemic and non-ischemic segments demonstrated that a cut-off value of -20% of stress LS had 71% sensitivity and 60% specificity for ruling out inducible myocardial ischemia (Area under the curve was AUC = 0.72, *P* < 0.0001).

**Conclusion:**

Myocardial LS measured with treadmill contrast-enhanced stress echocardiography demonstrates potential value in identifying patients with inducible myocardial ischemia.

## Introduction

Stress echocardiography (SE) is an important non-invasive imaging modality for diagnosing coronary artery disease (CAD) in patients presenting with chest pain. In comparison to nuclear perfusion imaging, SE has a similar sensitivity (88%) but a higher specificity (83%) in detecting coronary stenosis of greater than 50% [1, 2].

In the presence of significant coronary stenosis, exercise increases heart rate and results in myocardial oxygen demand-supply mismatch leading to a reduction in myocardial perfusion and metabolic alterations followed by regional wall motion abnormalities, ischemic ECG changes, and anginal symptoms [3].

Visual assessment of myocardial wall thickening, and endocardial excursions is the most common method used to assess regional wall motion during SE interpretation [1, 3]. The presence of new and/or worsening wall motion abnormalities is suggestive of reproducible myocardial ischemia. Moreover, left ventricle (LV) cavity dilatation at peak exercise, typical ST-segment changes, and ventricular arrhythmias are often indicative of ischemia [3].

Respiratory-related motion artifacts and ultrasound dropouts can compromise image quality and SE diagnostic accuracy. thus, ultrasound enhancing agents (UEA) are being increasingly used to improve endocardial border delineation and interpretation accuracy of SE [3].

Peak systolic global longitudinal strain (GLS) derived from speckle-tracking echocardiography is the most commonly used strain parameter to evaluate myocardial contractility and to define subtle myocardial changes [4]. GLS is extensively investigated in myocardial diseases such as amyloidosis and cancer chemotherapy-related cardiotoxicity [5, 6]. A few reports have analyzed the role of GLS with dobutamine SE and found that longitudinal strain can improve the diagnostic accuracy of SE [7–9]. A paucity of data is available about using GLS with treadmill SE likely due to the interference of respiratory motion artifacts with endocardial tracing [10].

UEA facilitates accurate and reproducible GLS analysis in approximately 94% of the contrast-enhanced scans, even in patients with poor or suboptimal nonenhanced image quality [11]. However, there is a lack of studies investigating the role of GLS measurements using enhanced echocardiography with treadmill SE. Therefore, this study aims to analyze the value of GLS in diagnosing myocardial ischemia in patients with chest pain undergoing treadmill contrast-enhanced stress echocardiography.

## Methods

### Patient selection

We retrospectively enrolled all patients who were referred from a dedicated chest pain clinic for treadmill stress echocardiography, and subsequently underwent coronary angiography between January 2020 to December 2021. All patients signed an informed consent to be enrolled in the chest pain study. The study protocol was approved by our institutional ethics committee. Patients with previous coronary artery disease or coronary revascularization, who did not reach the age-predicted target heart rate and patients with poor image quality for GLS assessment were excluded.

### Echocardiography

At rest, standard two-dimensional enhanced and non-enhanced cine-loops were recorded in apical 4-chamber (CH), 3-CH, and 2-CH as well as parasternal long-and short-axis views. UEA (Definity, Lantheus Medical Imaging, North Billerica, Massachusetts, 1.5 mL of 1.5 mL Definity in 10 mL saline over 10 s) was injected 30 s before the termination of the stress test. Additional 1 mL doses of UEA were subsequently administered to ensure optimal left ventricular cavity opacification. Similar enhanced cine-loops were taken within 60–90 s immediately after exercise and then 3–4 min for after recovery images.

### Treadmill exercise

A standard Bruce exercise protocol was used for all patients, with blood pressure, heart rate, and continuous electrocardiographic monitoring performed during the stress test. To improve the sensitivity of the test, patients were encouraged to continue exercising after achieving > 85% of the maximum age-predicted target heart rate (MAHR) which calculated using the formula: MAHR= (achieved heart rate /220-age) x 100. Criteria used for test termination included significant ST-segment changes, disabling symptoms (chest pain dyspnea, and dizziness), or significant arrhythmia. Exaggerated blood pressure response to exercise was defined as peak systolic blood pressure > 220 mmHg [3].

### Coronary angiography

All patients underwent invasive coronary angiography using standard procedural techniques. Multiple projections to assess the coronary arteries were performed. The degree of coronary stenosis was initially visually assessed and considered significant if luminal stenosis of more than 70%. Where in doubt fractional flow reserve (FFR) of < 0.8 was used to assess the severity of stenosis.

### Echocardiography interpretation

An EPIQ cardiac ultrasound machine (Philips Medical Systems, Andover, Massachusetts) was used on all patients. Definity (Lantheus Medical Imaging, North Billerica, Massachusetts) was used as UEA during rest, peak-stress and recovery acquisition to improve left ventricle opacification and endocardial border definition. All echocardiographic views were transferred to a dedicated software program for off-line assessment. Rest, peak-stress and recovery views were aligned and assessed for the presence of new and/or worsening of regional wall motion, abnormal myocardial thickening, LV cavity size dilatation and for strain imaging analysis with Image TomTec Arena software (Ultrasound Workspace Lot 50).

### LS interpretation

**Fig. 1:**
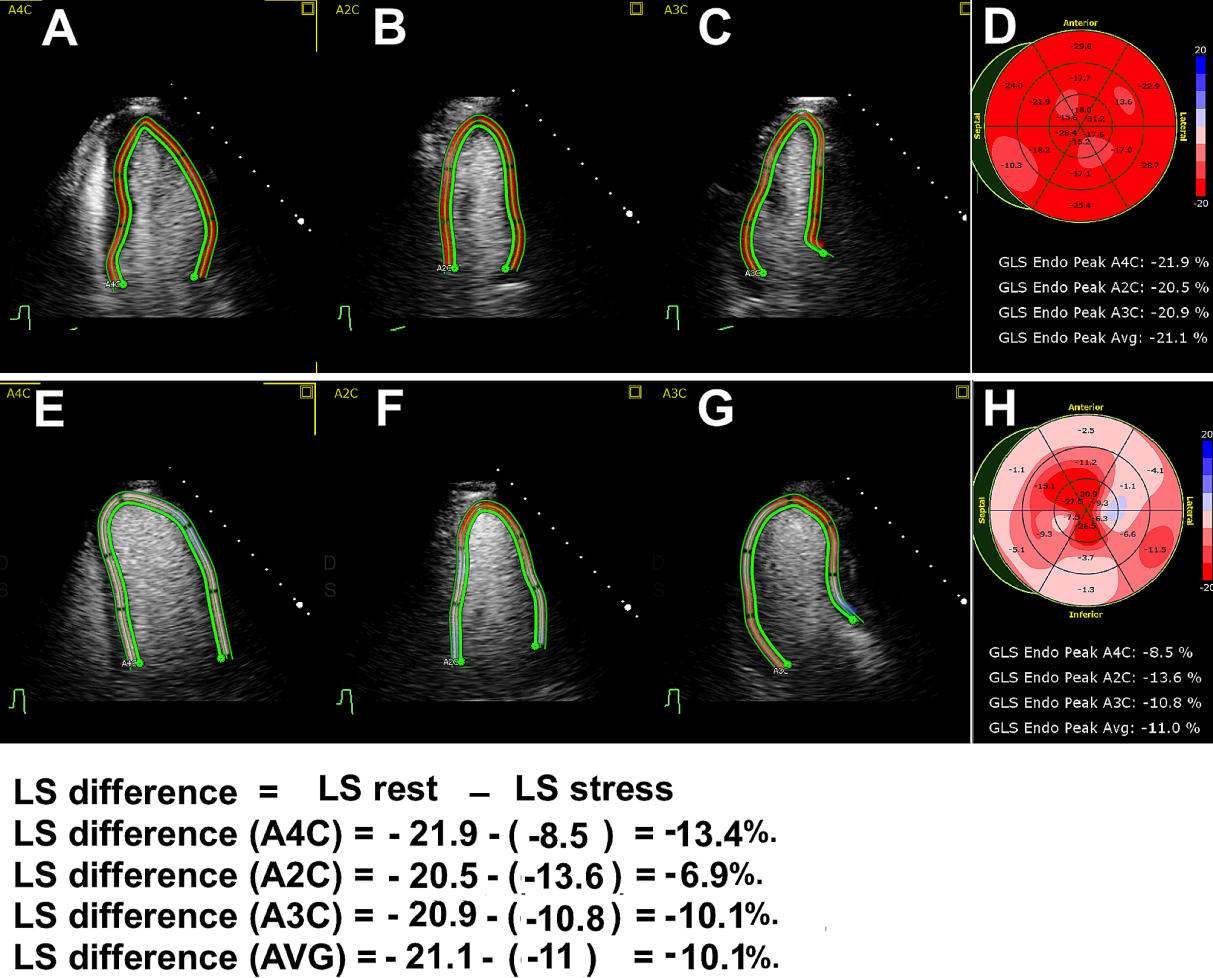
The top row illustrates rest strain imaging in a patient with three-vessel disease, **A**: Apical 4-chamber (4-CH), **B**: Apical 2-CH, **C**: Apical 3-CH view and **D**: Bull’s eye strain map of left ventricular segments shows mild segmental strain reduction at rest; however, the averaged 4-CH, 2CH, 3-CH and averaged global longitudinal strain was normal The bottom row illustrates peak stress strain in the same patient, **E**: Apical 4-chamber (4-CH), **F**: Apical 2-CH, **G**: Apical 3-CH view and H: Bull’s eye strain map of left ventricular segments shows severe reduction of the segmental and averaged strain reduction

LS measurement was done with 2D Speckle tracking. readers who measured the LS were blinded to angiography results. We used the contrast-enhanced apical 2-CH, 3-CH and 4-CH views for LS measurement at rest and at peak exercise. Manual editing of the endocardial border tracing was performed if necessary. We reported the LS of each myocardial segment, the averaged GLS of the entire myocardium, GLS of the 4-, 3- and 2-chamber views (resting GLS value more negative than (-18%) is considered as definitely normal). LS values of each myocardial wall (anterior, anterolateral, inferolateral, inferior, inferoseptal, and anteroseptal) were obtained by averaging the basal, mid, and apical LS of each wall. Figure 1.

We also defined the differences in LS as follow: LS difference = LS rest - LS stress.

To compare LS and coronary angiography results, we correlated LV segments with the vascular distribution as per practice guidelines [12]. Specifically, mid and basal segments of the inferior and inferoseptal segments related to the right coronary artery territory (RCA). The mid and basal lateral and anterolateral segments are considered related to circumflex coronary artery (LCX) territory. The remaining myocardial segments of the 16-segment model were considered to fall within the distribution of the left anterior descending coronary artery (LAD) territory.

### Intra- and inter-observer agreement

In order to evaluate both intra- and inter-observer variations, we randomly selected 25 rest and 25 stress scans. One reader performed repeated strain measurements to assess intra-observer variation, while two different readers were tasked with obtaining strain measurements to assess inter-observer variability.

### Statistical analysis

We performed the Mann-Whitney U test to compare GLS between patients with significant and non-significant coronary artery stenosis, while the McNemar test was used to compare dichotomous variables between the two groups. We performed a Receiver-operating characteristic (ROC) analysis of the ischemic and non-ischemic myocardial segments to evaluate the diagnostic accuracy of abnormal GLS on diagnosing myocardial ischemia. Statistical Package for Social Sciences (SPSS 22) was used for data analysis.

## Results

### Baseline characteristics

In total, 36 patients were enrolled. LS measurement with contrast-enhanced echocardiography was feasible in 33/36 (91.6%), while 3 patients (8.4%) were excluded due to poor image quality and unsuccessful GLS measurement. The mean age was (64.8 ± 11) years, and 20 (60.6%) were men. Patients with non-critical stenosis had a significantly higher prevalence of diabetes than patients with coronary stenosis. Other baseline characteristics did not differ between groups, Table 1.

**Table 1:** Patients’ baseline characteristics

Variables	Coronary artery stenosis (*N* = 16)	No coronary artery stenosis (*N* = 17)	*P* value
Age (years), mean ± SD	64.9 ± 12	64.7 ± 11	0.9
Achieved MAHR (%), mean ± SD	93 ± 8	102 ± 7	0.004
Gender - male n (%)	8 (50%)	12 (70.6%)	0.6
Hypertension, n (%)	10 (62.5%)	10 (58.8%)	0.45
Dyslipidemia, n (%)	9 (56.3%)	7 (41.2%)	0.9
Diabetes Mellitus, n (%)	0 (0%)	2 (11.8%)	0.001
Smoking, n (%)	6 (37.7%)	4 (23.5%)	0.18
Family history of coronary artery disease, n (%)	5 (31.3%)	9 (52.9%)	0.82
Hypertensive response n (%)	1 (6.3%)	3 (17.6%)	0.008
Electrocardiogram changes n (%)	9 (56.3%)	8 (47.1%)	0.85

MAHR: Maximum age-predicted heart rate (%)

### Stress test results

All patients underwent treadmill stress test, with the achieved MAHR and exaggerated systolic hypertension response notably higher in patients with non-critical coronary artery stenosis (102% ± 7 vs. 93% ±8, *P* = 0.004 and 6.3% vs. 17.6%, *P* = 0.008).

### Coronary angiography results

We found 16/33 (48.4%) patients had significant coronary artery stenosis and underwent revascularization. In detail: One patient had three-vessel disease, two patients had two-vessel disease, and 13 patients had single vessel disease (seven patients had LAD stenosis, and six patients had RCA stenosis).

### Myocardial strain results

At rest, longitudinal strain values did not differ between patients with and without critical coronary artery stenosis as follow: Averaged GLS (-22 ± 5 vs. -22 ± 6, *p* = 0.61), 2-CH (− 22 ± 8 vs. -22 ± 6, *p* = 0. 43), 3-CH (-20 ± 3 vs. -22 ± 4, *p* = 0.79), and 4-CH (-21 ± 6 vs. -21 ± 3, *p* = 0.65), respectively.

**Fig. 2:**
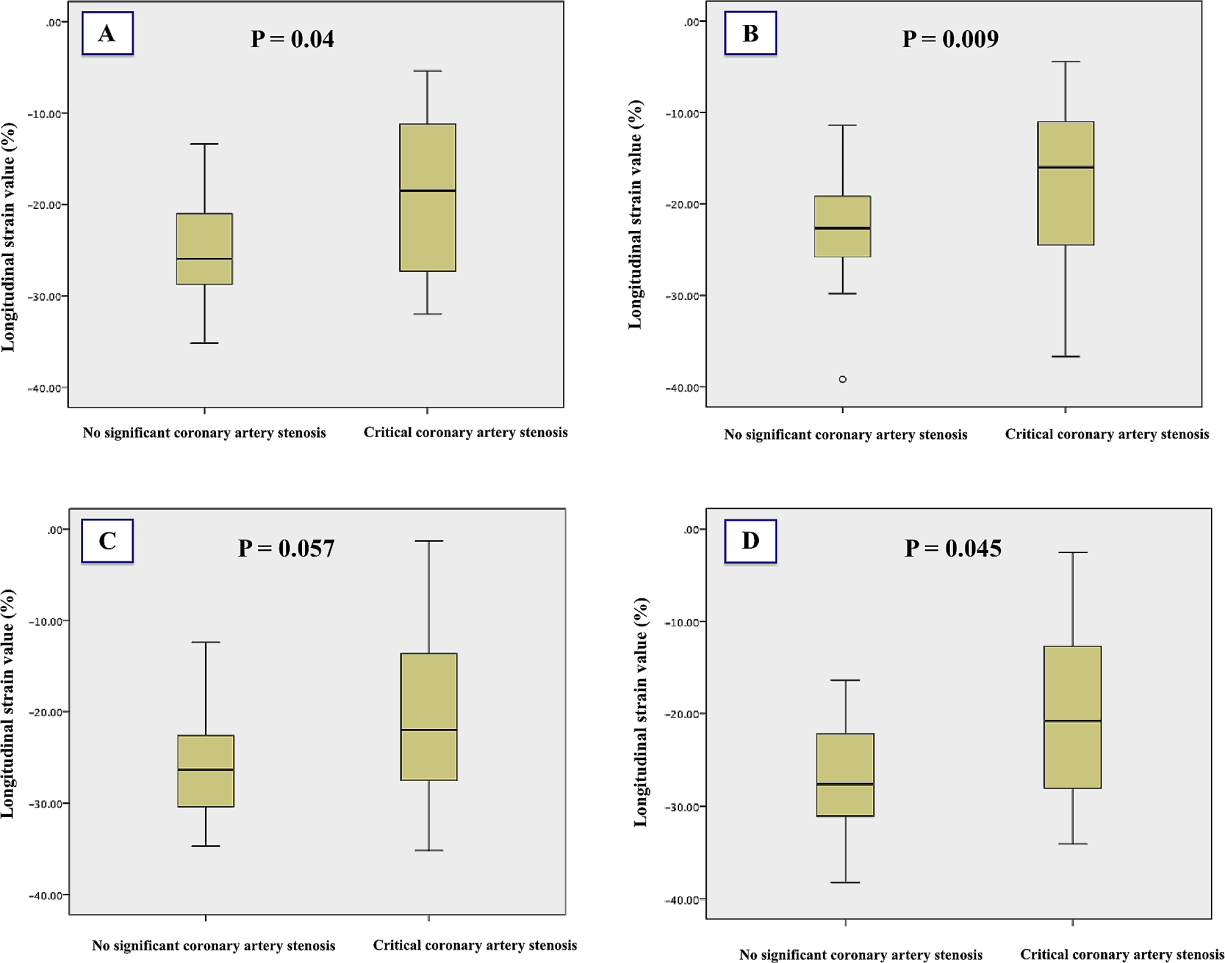
Graph demonstrates (**A**): Averaged global longitudinal strain, (**B**): Four-chamber, (**C**): Two-, and (**D**): Three-chamber longitudinal strain at peak stress in patients with and without critical coronary artery stenosis

At peak stress, the averaged GLS, 3-CH, and 4-CH longitudinal strain values were significantly less negative in patients with coronary artery stenosis when compared to patients with non-significant stenosis: (-17 ± 7 vs. -24 ± 7, *p* = 0.041), (-18 ± 9 vs. -25 ± 8, *p* = 0.045) and (-15 ± 6 vs. -23 ± 8, *p* = 0.009), respectively, Fig. 2. Other myocardial strain values are shown in Table 2.

**Table 2:** Averaged stress GLS and stress longitudinal strain values of left ventricle segments, and walls in patients with and without severe coronary artery stenosis

LS of myocardial segments and walls at stress (%), mean ± SD	Coronary artery stenosis (*N* = 16)	No coronary artery stenosis (*N* = 17)	*P* value
Basal anterior	-18 ± 11	-22 ± 10	0.2
Mid anterior	-20 ± 14	-23 ± 11	0.2
Apical anterior	-18 ± 15	-28 ± 9	0.032
Basal lateral	-14 ± 11	-20 ± 12	0.11
Mid lateral	-14 ± 13	-19 ± 8	0.14
Apical lateral	-17 ± 10	-26 ± 11	0.02
Basal posterior	-18 ± 10	-24 ± 9	0.14
Mid Posterior	-23 ± 13	-23 ± 9	0.5
Apical posterior	-24 ± 13	-27 ± 11	0.4
Basal inferior	-16 ± 10	-21 ± 11	0.15
Mid inferior	-19 ± 11	-22 ± 11	0.23
Apical inferior	-24 ± 13	-27 ± 12	0.29
Basal inferoseptal	-12 ± 8	-18 ± 10	0.08
Mid inferoseptal	-14 ± 7	-22 ± 9	0.023
Apical inferoseptal	-15 ± 11	-23 ± 12	0.02
Basal anteroseptal	-12 ± 11	-18 ± 10	0.14
Mid anteroseptal	-14 ± 12	-21 ± 9	0.03
Apical anteroseptal	-23 ± 17	-32 ± 13	0.05
Two-Chamber	-18 ± 9	-25 ± 8	0.057
Three-Chamber	-18 ± 9	-25 ± 8	0.04
Four-Chamber	-15 ± 6	-23 ± 8	0.009
Averaged	-17 ± 7	-24 ± 7	0.041
Anterior	-19 ± 9	-24 ± 8	0.03
Lateral	-15 ± 9	-21 ± 8	0.08
Posterior	-22 ± 10	-25 ± 6	0.41
Inferior	-20 ± 10	-23 ± 9	0.19
Inferoseptal	-14 ± 6	-20 ± 7	0.016
Anteroseptal	-16 ± 9	-24 ± 8	0.047

GLS: Global longitudinal strain

Similarly, patients with critical coronary artery stenosis had lower LS-difference values than patients with non-significant stenosis as follow: Averaged GLS-difference was (-6 ± 6 vs. 2 ± 6, *p* = 0.003), 2-CH LS-difference was (-5 ± 8 vs. 2 ± 8, *p* = 0.031), 3-CH LS-difference was (-5 ± 8 vs. 5 ± 8. *p* = 0.009), and 4-CH LS-difference was (-8 ± 6 vs. 1 ± 7, *p* = 0.001), respectively, Table 3.

**Table 3:** Longitudinal strain difference of the left ventricle walls and the averaged GLS differences in patients with and without severe coronary artery stenosis

LS stress – LS rest	Coronary artery stenosis *N* = 16	No coronary artery stenosis *N* = 17	*P* value
Two-Chamber	− 5 ± 8	2 ± 8	0.03
Three-Chamber	− 5 ± 8	5 ± 8	0.009
Four-Chamber	− 8 ± 6	1 ± 7	0.001
Averaged LGS	− 6 ± 6	2 ± 6	0.003
Anterior wall	− 5 ± 9	2 ± 8	0.051
Lateral wall	− 7 ± 10	− 1 ± 8	0.089
Posterior wall	0.3 ± 11	3 ± 8	0.53
Inferior wall	0 ± 10	1 ± 10	0.13
Infero-septal wall	− 7 ± 5	0 ± 7	0.018
Anteroseptal wall	1 ± 10	4 ± 8	0.02

GLS: Global longitudinal strain

**Fig. 3:**
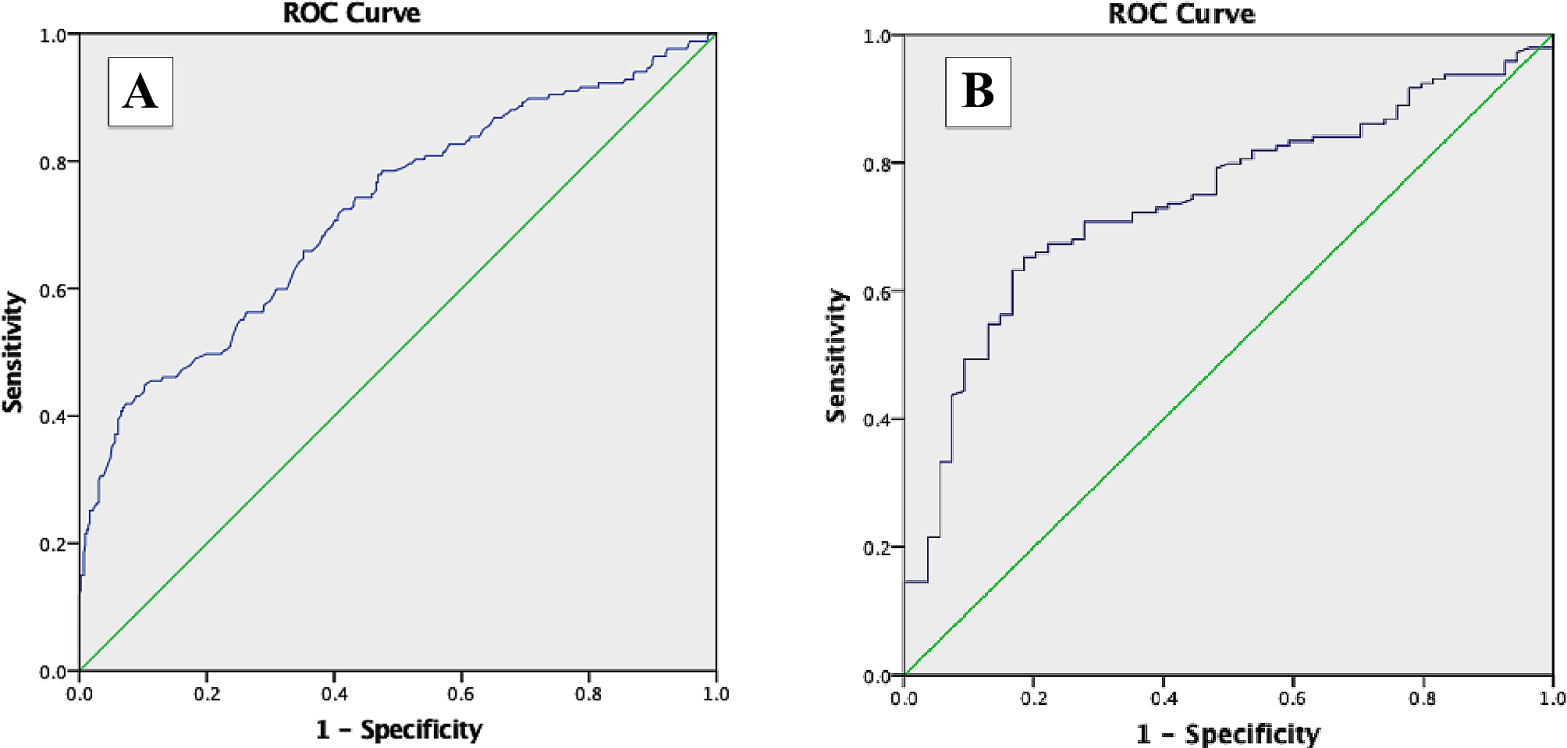
Receiver operating characteristic (ROC) curve for identifying the sensitivity and specificity of (**A**) stress longitudinal strain, and (**B**) longitudinal strain difference in detecting inducible myocardial ischemia

Receiver operating characteristic curve analysis of the ischemic and non-ischemic segments demonstrated that a cut-off value of -20% of stress LS had 71% sensitivity and 60% specificity to rule out inducible myocardial ischemia (Area under the curve was AUC = 0.72, *P* < 0.0001), while a cut-off value of -3% LS difference score yielded 71% sensitivity and 72% specificity for ruling out inducible myocardial ischemia (Area under the curve was AUC = 0.74, *P* < 0.0001), Fig. 3. In this study we found a very good inter-observer and intra-observer agreement for LS measurements (0.89 and 0.87, respectively).

## Discussion

To our knowledge, this is the first study investigating the utility of LS with contrast-enhanced echocardiography for diagnosing myocardial ischemia during treadmill SE. Our findings reveal that patients with critical coronary stenosis exhibit significantly lower stress 2-CH, 4-CH, 3-CH and averaged LS compared to those with non-critical coronary stenosis. Furthermore, a stress LS of less negative than − 20% demonstrates a sensitivity of 71% and specificity of 60%, while LS difference of more negative than 3% has sensitivity of 71% and specificity of 72% in identifying inducible myocardial ischemia.

Although qualitative assessment is not recommended in the evaluation of LV systolic function in rest echocardiographic studies, visual interpretation remains the standard method for evaluating myocardial excursion during SE, and that may result in inter- and intra-observer variations during scan interpretation [13]. Notably, Picano et al. [14] demonstrated that SE interpretation is operator-dependent, with a lower diagnostic accuracy observed among readers with limited experience, namely those who have performed less than one hundred SE scans.

Cadeddu et al. [7] investigated the role of LS in identifying myocardial ischemia in 20 patients using non-enhanced echocardiography and found that LS had a sensitivity of 90% and specificity of 93% in detecting ischemia with dobutamine SE. Similarly, Wierzbowska-Drabik et al. [15] demonstrated that averaged GLS during dobutamine SE accurately differentiates hypokinetic from normo-kinetic myocardial segments at rest as well as at the peak exercise. The authors also showed that LS reduction at peak stress is more predominant in segments with impaired contractility when compared to those with visually normal kinetic [15]. Bjork Ingul et al. compared the diagnostic accuracy of strain imaging versus visual assessment in the detection of myocardial ischemia with non-enhanced dobutamine stress echocardiography, concluding that strain assessment had higher sensitivity but similar specificity, and overall diagnostic accuracy in comparison to visual assessment [16].

In this study, we found that strain imaging with UEA was feasible in 91.7% of the stress scans, consistent with a previous report by Medvedofsky et al. who demonstrated that LS analysis is applicable in 94% of the rest enhanced-echocardiographic studies [11]. Moreover, the authors also found a good agreement between GLS measured with contrast and non-contrast, as well as CMR-derived strain with *r* = 0.85 and *r* = 0.83, respectively.

There is a widespread agreement that artificial intelligence has the potential to transform diagnostic medical imaging by improving diagnostic accuracy, shortening reading time, and reducing inter-observer variations. Our study illustrates the importance of utilizing machine learning in the diagnosis of myocardial ischemia using LS, further efforts to propose a deep learning-based model co-registers LS results with patients’ demographics, stress test, and ECG data to improve the test outcome is warranted [17].

This study has a few limitations. Firstly, it is a retrospective analysis with a relatively small number of patients were enrolled highlighting the need for larger prospective studies for validation. Secondly, strain measurements may be influenced by various factors, including changes in afterload and preload during stress, which may confound the interpretation of results.

Thirdly, the software used for analysis was dedicated to non-enhanced echocardiographic scans, necessitating manual editing of the endocardial borders in most patients. Lastly, given the vendor variations GLS assessment, the results of our study cannot be extrapolated to other vendor software.

## Conclusion

Our study demonstrates that myocardial LS measurements utilizing contrast-enhanced echocardiography is feasible in approximately 92% of patients undergoing SE. Furthermore, our findings underscore the pivotal role of stress LS and LS difference. These findings highlight the potential clinical utility of incorporating LS evaluation into SE protocols for improved detection and management of ischemic heart disease.

## Data Availability

The data that support the findings of this study are available from the corresponding author upon reasonable request.

## Abbreviations

CAD: Coronary artery disease
GLS: Global longitudinal strain
LAD: Left anterior descending coronary artery
LCX: Circumflex coronary artery
LS: Longitudinal strain
RCA: Coronary artery territory
SE: Stress echocardiography
MAHR: Maximum age-predicted target heart rate
