# Correction: Role of myocardial strain imaging in diagnosing inducible myocardial ischemia with treadmill contrast-enhanced stress echocardiography

**DOI:** 10.1186/s12872-025-04657-0

**Published:** 2025-03-15

**Authors:** Rami M. Abazid, Nilkanth Patil, Maged Elrayes, Mark Chandy, Magdi Hassanin, Andrew Mathew, Sabe De, Rodrigo Bagur, Nikolaos Tzemos

**Affiliations:** 1 Northern Ontario Medical School (NOSM) University, Department of Medicine, Sault Area Hospital, Sault Ste. Marie, Canada; 2 Division of Cardiology, Department of Medicine, London Health Sciences Centre, University Hospital, PO Box 5010, 339 Windermere Road, London, ON N6A 5A5 Canada

**Correction: BMC Cardiovasc Disord 24, 254 (2024)**

**https://doi.org/10.1186/s12872-024–03926-8**

In this article [1], the author’s name Nilkanth Patil was incorrectly written as Nilkanth Pati.

The original article has been corrected.
